# Newly imported proteins in mitochondria are particularly sensitive to aggregation

**DOI:** 10.1111/apha.13985

**Published:** 2023-06-01

**Authors:** Carmela Vazquez‐Calvo, Verena Kohler, Johanna L. Höög, Sabrina Büttner, Martin Ott

**Affiliations:** ^1^ Department of Biochemistry and Biophysics Stockholm University Stockholm Sweden; ^2^ Department of Molecular Biosciences, The Wenner‐Gren Institute Stockholm University Stockholm Sweden; ^3^ Institute of Molecular Biosciences University of Graz Graz Austria; ^4^ Department of Chemistry and Molecular Biology University of Gothenburg Gothenburg Sweden; ^5^ Department of Medical Biochemistry and Cell Biology University of Gothenburg Gothenburg Sweden

**Keywords:** aggregates, aging, cellular stress, Hsp78, metabolism, mitochondria, protein quality control, proteostasis

## Abstract

**Aim:**

A functional proteome is essential for life and maintained by protein quality control (PQC) systems in the cytosol and organelles. Protein aggregation is an indicator of a decline of PQC linked to aging and disease. Mitochondrial PQC is critical to maintain mitochondrial function and thus cellular fitness. How mitochondria handle aggregated proteins is not well understood. Here we tested how the metabolic status impacts on formation and clearance of aggregates within yeast mitochondria and assessed which proteins are particularly sensitive to denaturation.

**Methods:**

Confocal microscopy, electron microscopy, immunoblotting and genetics were applied to assess mitochondrial aggregate handling in response to heat shock and ethanol using the mitochondrial disaggregase Hsp78 as a marker for protein aggregates.

**Results:**

We show that aggregates formed upon heat or ethanol stress with different dynamics depending on the metabolic state. While fermenting cells displayed numerous small aggregates that coalesced into one large foci that was resistant to clearance, respiring cells showed less aggregates and cleared these aggregates more efficiently. Acute inhibition of mitochondrial translation had no effect, while preventing protein import into mitochondria by inhibition of cytosolic translation prevented aggregate formation.

**Conclusion:**

Collectively, our data show that the metabolic state of the cells impacts the dynamics of aggregate formation and clearance, and that mainly newly imported and not yet assembled proteins are prone to form aggregates. Because mitochondrial functionality is crucial for cellular metabolism, these results highlight the importance of efficient protein biogenesis to maintain the mitochondrial proteome operational during metabolic adaptations and cellular stress.

## 1 INTRODUCTION

The maintenance of a functional proteome is a critical task for cells and relies on the activities of sophisticated protein quality control (PQC) systems in the cytosol and organelles. A functional decline of PQC systems is associated with aging and age‐associated diseases, resulting in the accumulation of misfolded proteins and their sequestration into protein aggregates.^1^, ^2^ Like the PQC system in the cytosol, mitochondria are equipped with sets of molecular chaperones to support de novo protein folding, aggregate handling factors like protein unfoldases, and proteases for general and targeted protein removal.^3^ Mitochondria play essential roles in energy metabolism, cofactor biosynthesis, lipid and amino acid metabolism, and the regulation of programmed cell death.^4^ Mitochondrial oxidative phosphorylation (OXPHOS) allows eukaryotic cells to use a highly efficient way of energy conversion. Here, energy from nutrients is converted to ATP by using oxygen as the terminal electron acceptor. Cells can use various metabolic pathways to exploit certain energy sources. In the case of glucose utilization, also referred to as fermentation, mitochondrial energy conversion is repressed, but the organelle still serves as important hub for intermediate metabolism.^5^ To execute all these diverse biochemical functions, mitochondria contain around 1000 different proteins,^6^, ^7^ most of which are encoded by nuclear genes. Thus, the majority of the mitochondrial proteome is synthesized in the cytosol to be imported into the organelle, which relies on highly conserved import machineries.^8^ Proteins destined for the mitochondrial matrix mostly carry cleavable mitochondrial targeting sequences^9^ that are recognized by receptors on the mitochondrial outer membrane.^10^ These precursor proteins are then threaded through the translocases in the outer and inner membranes to finally reach the mitochondrial matrix, where chaperones of the Hsp70 family support their translocation and subsequent folding.^11^ Many mitochondrial proteins are part of larger complexes and thus require further assembly steps to reach the final function. Maintenance of a functional mitochondrial proteome relies on the mitochondrial PQC system, including molecular chaperones, aggregate handling factors, as well as proteases, many of which are similar to their bacterial ancestors. Expression of this PQC is not only partly under control of the general heat shock response, but also part of the general activation of mitochondrial biogenesis.^12^

Recent work has demonstrated that during aging and upon exposure to environmental stress, protein aggregates form in the cytosol and the mitochondrial matrix.^13^, ^14^, ^15^ Here, ClpB‐type unfoldases like the cytosolic Hsp104 and its mitochondrial homolog Hsp78 decorate these aggregates and dissolve them through extraction of individual polypeptides.^16^, ^17^, ^18^, ^19^ Hsp78 contains, like the other ClpB homologs, a substrate binding domain as well as an associated AAA‐type of ATPase, which is used to unfold a bound protein by threading through a central pore.^20^ Due to the interdependency between cytosolic and mitochondrial metabolism, the metabolic state of the cell likely determines the efficiency with which the mitochondrial PQC system operates.^21^ Moreover, it remained unclear which proteins aggregate within mitochondria and how mitochondria handle and clear these aggregates.

## 2 RESULTS

### 2.1 Mitochondrial aggregate formation under stress and aging

**FIGURE 1:**
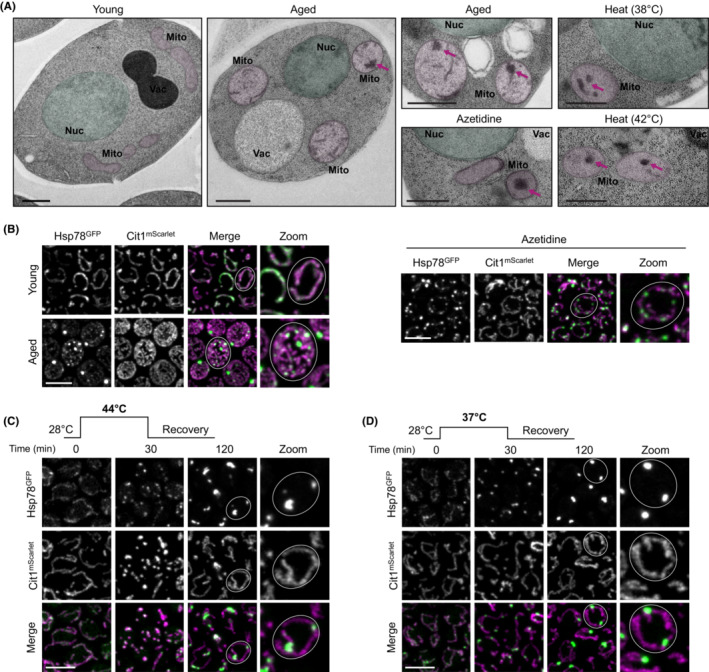
Mitochondrial aggregate formation under stress and aging. (A) Transmission electron micrographs of wild‐type cells at different growth stages (young—8 h, aged—48 h) and upon treatment with different stressors: 1 mg/mL azetidine or heat for 45 min at 38°C and 30 min at 42°C. Nucleus (Nuc), vacuole (Vac), and mitochondria (Mito) are labeled. Arrows point at mitochondrial aggregates. Scale bars: 500 nm. (B) Confocal micrographs of Hsp78^GFP^ localization under the same conditions as in (A). Scale bar: 5 μm. Confocal micrographs of the mitochondrial network (Cit1^mScarlet^) and Hsp78^GFP^ localization to aggregates upon exposure to heat stress at 44°C (C) or 37°C (D) for 30 min as well as 90 min after the stress (recovery). Brightness and contrast were independently adjusted at 0 min to ensure the visualization of the mitochondrial network. Scale bar: 5 μm.

Protein aggregates within mitochondria form during aging and in response to environmental stress across species.^14^, ^15^, ^22^, ^23^, ^24^ However, while mechanisms of aggregate handling in the cytosol are well understood, it is less clear how mitochondria deal with protein aggregates. In yeast cells, mitochondrial protein aggregates, visible as large, electron‐dense clusters within the mitochondrial matrix in electron micrographs, form during cellular aging, upon treatment with azetidine, a proline analog that induces protein misfolding, as well as after heat stress (Figure 1A). To shed light on the dynamics of aggregate formation and clearance in mitochondria, we followed a GFP‐tagged version of Hsp78 via confocal microscopy. Hsp78 is a member of the ClpB‐type unfoldases, which can extract misfolded proteins from aggregates with the virtue of its ATPase domain. Hsp78^GFP^ in logarithmically growing cells (Young) was evenly distributed throughout the mitochondrial network (visualized via Cit1^mScarlet^), but formed foci in stationary phase cells (Old) (Figure 1B), reminiscent of foci observed in previous studies employing Hsp78^14^, ^15^ or its cytosolic homolog, Hsp104,^18^ and the aggregates identified in electron microscopy (Figure 1A). Likewise, exposure to azetidine led to foci formation of Hsp78^GFP^ (Figure 1B). Next, we exposed cells grown at 28°C under fermentative conditions to a 30 min heat shock, after which the cells were transferred back to 28°C. Specifically, we used a severe (44°C) and a mild (37°C) heat shock to induce protein aggregation (Figure 1C,D). While Hsp78^GFP^ was evenly distributed before the heat shock, most of the Hsp78^GFP^ signal was found in multiple, distinct foci after heat shock. Following Hsp78^GFP^ up to 90 min after the heat shock showed that many of these aggregates were stable and did not dissolve. Interestingly, the severe heat shock at 44°C provoked a collapse of the mitochondrial network as judged from Cit1^mScarlet^ (Figure 1C), while the network remained intact after a heat shock at 37°C (Figure 1D). This is in line with a previous study showing a prominent mitochondrial fragmentation after severe heat shock,^25^ which likely reflects a severe cellular insult. Thus, we employed the milder heat shock at 37°C as a model to study aggregate handling, because we expected that under these conditions, cellular functionality could still allow clearance of the mitochondrial aggregates over time.

### 2.2 The metabolic state defines the handling of mitochondrial aggregates

**FIGURE 2:**
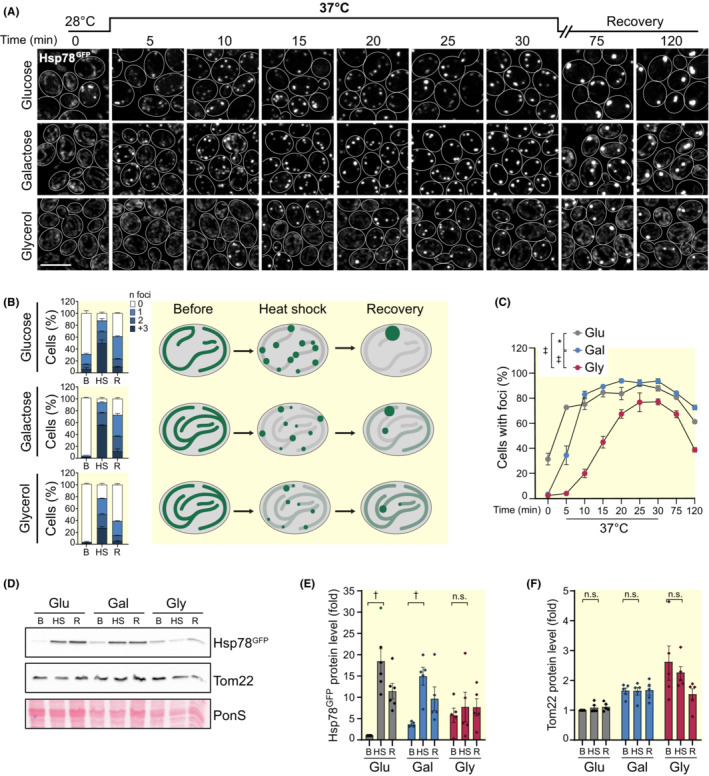
The metabolic state defines the handling of mitochondrial aggregates. (A) Timeline of mitochondrial aggregation dynamics in cells grown in media with different carbon sources (glucose, galactose, and glycerol). Hsp78^GFP^ was monitored every 5 min during heat shock at 37°C as well as 45 and 90 min after the stress (recovery phase). Brightness and contrast were independently adjusted (0–20 min), to ensure proper visualization of the mitochondrial network and aggregates. Scale bar: 5 μm. (B) Quantification of the number of aggregates per cell presented in (A) at time points 0, 30, and 120 min; *n* ≥ 110 cells were quantified per condition per time point. A model of mitochondrial aggregation dynamics under the different metabolic conditions is shown. (C) Quantification of the number of cells with aggregates during the timeline in (A). Error bars represent standard error of the mean (SEM); *n* ≥ 110 cells were quantified per condition per time point. (D–F) Immunoblot analysis of protein extracts before [B], after a 30‐min heat shock at 37°C [HS] and 90 min during recovery [R]. Representative blots (D) and quantifications (E,F) are shown. Membranes were probed with antibodies against GFP and Tom22. Ponceau S (PonS) staining was used as loading control and protein levels were normalized to glucose before heat shock (B‐Glu). Individual values and SEM are depicted; *n* = 5 (^n.s.^ *p* > 0.05; *p < 0.05; ^†^ *p* < 0.01; ^‡^ *p* < 0.001).

Next, we asked how the cellular metabolic state would influence mitochondrial aggregate handling. The mitochondrial ATP content depends on cellular metabolism, and chaperones involved in protein refolding like the Hsp70, chaperonins like HSP60, or unfoldases like Hsp78 depend on ATP to power their activities.^21^ Likewise, steady‐state levels of the chaperones in mitochondria are determined by specific gene expression programs, which are activated or repressed depending on the physiological state of the cell. While genes involved in mitochondrial function and biogenesis are repressed during glucose fermentation, they are induced during respiratory growth.^5^ To analyze aggregate handling depending on the metabolic state, cells were grown in different carbon sources, namely glucose to permit fermentation, glycerol, which is metabolized by respiration, and galactose, which can be fermented, but does not provoke downregulation of respiration by glucose repression.^5^ Cells were exposed to a 30‐min heat shock at 37°C and the distribution of Hsp78^GFP^ was recorded. Multiple small mitochondrial aggregates were seen as early as 5 min after the start of the heat shock in cells grown on glucose (Figure 2A). In many cells, these aggregates persisted throughout the 2 hours of the experiment and coalesced into a single, large aggregate per cell during later stages of the chase (Figure 2A,B). Cells grown on galactose started to form aggregates later but were also unable to clear them (Figure 2A,B). In contrast, cells grown under purely respiratory conditions (glycerol) were most resistant to aggregate formation and cleared the generated aggregates in the recovery phase quite efficiently (Figure 2A,B). Automated quantification of cells with aggregates confirmed that a respiratory metabolism delays the onset of heat‐induced protein aggregation and supports protein disaggregation (Figure 2C). One possible explanation for this difference in aggregate handling could be differences in the expression levels of Hsp78. Indeed, respiring cells contained substantially higher levels of Hsp78 protein than cells fermenting glucose before the heat shock (Figure 2D,E). This was not simply due to increased mitochondrial mass in respiring cells, as the protein levels of Tom22, a subunit of the mitochondrial protein translocation machinery, did not increase to the same extent (Figure 2F). Interestingly, the difference in Hsp78 levels between the different metabolic states disappeared after the heat shock. Glucose fermenting cells prominently upregulated Hsp78 (Figure 2D,E), presumably via heat shock factor 1 (Hsf1)‐dependent signaling,^12^, ^26^ which likely also explains the increased intensity of Hsp78^GFP^‐positive foci observed via confocal microscopy (Figure 2A). In contrast, this response was absent in respiring cells, where Hsp78 levels were unaffected by heat shock (Figure 2D,E). Taken together, these data indicate that the metabolic state of the cell influences the dynamics of aggregate formation and clearance in mitochondria during a mild heat shock. While cells fermenting glucose have mitochondria that are less prepared to handle unfolded proteins and show significant aggregate formation already at early time points, respiring cells show a delay in aggregate formation and a substantial clearing of these aggregates.

### 2.3 Hsp78 interacts with unassembled mitochondrial proteins that are aggregation prone

**FIGURE 3:**
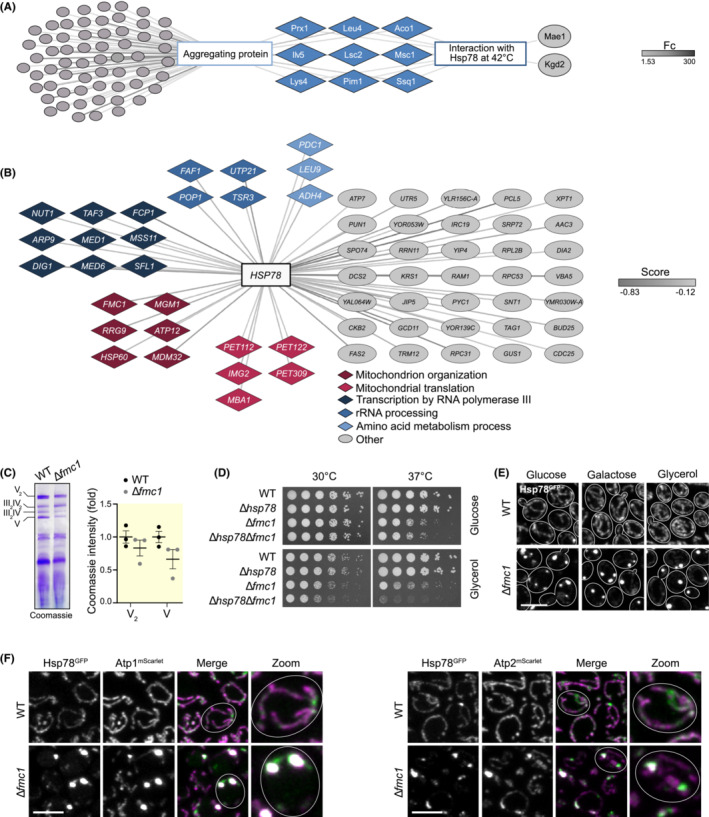
Hsp78 interacts with unassembled mitochondrial proteins that are aggregation prone. (A) Bioinformatic analysis of aggregation prone proteins that interact with Hsp78 under heat shock at 42°C (green). Data extracted from Jaworek et al.^27^ Connecting lines are quantitatively color‐coded according to the fold change (Fc). (B) Representation of Hsp78 significant negative genetic interactors (cutoff score < −0.12, *p* < 0.05). Data were extracted from TheCellMap and GO terms were imported via GO Slim Mapper from SGD. Groups are color‐coded based on their GO term and only those with at least four genes are shown. The rest are grouped under “Others.” (C) Blue native gel electrophoresis stained with Coomassie brilliant blue and quantification of the abundance of assembled ATP synthase monomer (V) and ATP synthase dimer (V_2_) in wild‐type and Δ*fmc1* cells; *n* = 3. (D) Drop dilution assay of WT cells (BY4742) and cells lacking Hsp78, Fmc1, or both in glucose‐ or glycerol‐containing media at 30 and 37°C. (E) Confocal micrographs of Hsp78^GFP^ localization in wild‐type and Δ*fmc1* cells grown in glucose, galactose, or glycerol. Scale bar: 5 μm. (F) Confocal micrographs of Hsp78^GFP^ and mScarlet‐tagged Atp1 or Atp2 in WT and Δ*fmc1* cells. Scale bar: 5 μm.

Previous studies have characterized both the interactome of Hsp78 and the composition of aggregates isolated from yeast cells after a heat shock.^27^, ^28^ To identify common hits and thereby get an understanding of the general propensities of aggregating proteins, we reanalyzed these datasets. This analysis revealed a common set of very abundant, soluble matrix proteins^6^ that are part of the aggregates and interact with Hsp78 upon heat stress (Figure 3A). As predicted for a general disaggregase, Hsp78 is primarily in contact with proteins that are also found in aggregates.

While Hsp78 is a general disaggregase, it might be particularly important under specific conditions, for instance, when a certain cellular pathway is compromised. Large, genome‐wide studies in yeast have revealed the interdependence of genes that modify individual phenotypes.^29^ Specifically, pairwise deletion of genes can either result in increased or decreased cellular fitness. A negative genetic interaction leads to an aggravation of the phenotype and could be explained by two redundant pathways being affected. Extracting these data from TheCellMap for *HSP78* revealed negative genetic interaction with several different mitochondrial pathways (Figure 3B). In particular, we were interested in proteins associated with general mitochondrial organization, such as chaperones (Figure 3B). In this cluster, mutants were retrieved that lacked proteins involved in either general protein folding, such as Hsp60, or playing roles as specific chaperones such as Atp12 and Fmc1, supporting ATP synthase assembly.^30^, ^31^ While Atp12 is absolutely required for ATP synthase assembly and its absence results in respiratory deficiency, Fmc1 is only important for ATPase assembly at higher temperatures.^31^ Specifically, Fmc1 facilitates a step in the assembly of the F1 part of ATP synthase,^31^ which is the matrix‐exposed part of the complex containing a hexameric assembly with alternating Atp1 and Atp2 subunits that mediate ATP formation. Indeed, substantial amounts of ATPase monomer and dimer could still be assembled in the absence of Fmc1 under normal temperature (Figure 3C). Thus, we decided to test its interdependency with Hsp78 in more detail and generated yeast mutants lacking Hsp78 and Fmc1 either individually or in combination. We performed serial dilution spotting assays to determine cellular fitness during fermentation or respiration (Figure 3D). As reported previously, cells grew robustly on respiratory media in the absence of Fmc1 at 30°C, but showed a growth defect under elevated temperature (Figure 3D). Interestingly, this respiratory growth defect was more prominent in a mutant lacking both Hsp78 and Fmc1 and already visible at 30°C (Figure 3D).

As the ATP synthase is a very abundant protein, defects in its assembly might result in aggregate formation of the non‐assembled Atp1 and Atp2 subunits already at normal temperature. Thus, we tested whether mitochondrial aggregates would form in cells lacking Fmc1. Indeed, we observed Hsp78‐containing foci in cells lacking Fmc1, irrespective of whether the cells were grown on fermentative or respiratory carbon source (Figure 3E). These foci indeed not only contained Hsp78 but also not‐assembled Atp1^mScarlet^ or Atp2^mScarlet^, respectively (Figure 3F). In sum, the genetic and functional interdependency of Hsp78 with ATP synthase biogenesis sheds light on how protein folding and assembly is linked to aggregate formation. It is likely that the reactivation of misfolded, aggregated Atp1 or Atp2 by Hsp78 is important to support their assembly in the absence of Fmc1, allowing mitochondrial function and respiratory growth.

### 2.4 Newly imported proteins are particularly prone to aggregation

**FIGURE 4:**
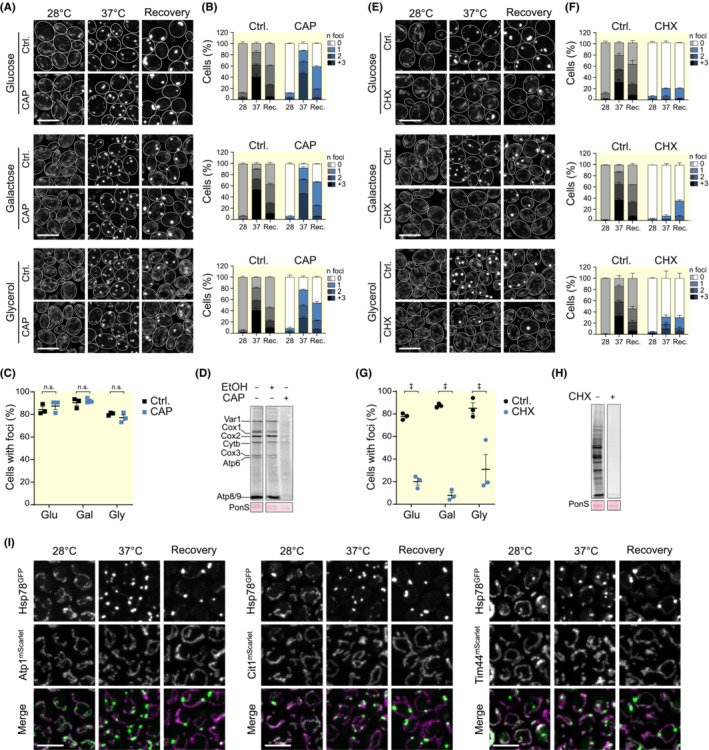
Effect of translation inhibitors on mitochondrial protein aggregation. (A) Representative confocal micrographs of Hsp78^GFP^ localization in cells grown in glucose, galactose, or glycerol, before (28°C), during (37°C), and 90 min after HS (recovery) with chloramphenicol (CAP) 1 mg/mL or solvent control. Brightness and contrast were adjusted independently (28°C) to ensure proper visualization of the mitochondrial network. Scale bar: 5 μm. (B) Quantification of the number of Hsp78^GFP^ foci per cell from cells described in (A); *n* ≥ 103 cells were quantified per condition per time point. (C) Comparison of the number of cells with Hsp78^GFP^‐decorated aggregates after heat shock (37°C) in cells described in (A). Mean (line) and SEM are depicted. (D) Autoradiogram of newly synthesized mitochondrial proteins in untreated cells or in the presence of CAP (1 mg/mL) or solvent control. (E) Representative micrographs of Hsp78^GFP^ mitochondrial distribution upon treatment with 150 μg/mL cycloheximide (CHX) or respective solvent control at the same time points as in (A). (F) Quantification of the number of Hsp78^GFP^ foci per cell in cells described in (E); *n* ≥ 88 cells per condition per time point. (G) Comparison of the number of cells with Hsp78^GFP^‐decorated aggregates after heat shock (37°C) in cells described in (E). Mean (line) and SEM are depicted. (H) Autoradiogram of newly synthesized proteins in the presence of CHX (150 μg/mL) or solvent control. (I) Confocal micrographs of Hsp78^GFP^ combined with mScarlet‐tagged Atp1, Cit1, or Tim44 at the same time points as (A) and (E). (^n.s.^ *p* > 0.05; ^‡^ *p* < 0.001).

These data pointed to a possible vulnerability of newly imported proteins, which could be prone to misfold before reaching their final fold and assembly. While mitochondrial translation produces mainly highly hydrophobic subunits of the oxidative phosphorylation system,^32^ cytoplasmic translation produces the main part of the mitochondrial proteome, of which a fraction requires assembly into OXPHOS complexes. Thus, we next asked whether aggregate formation was dependent on either cytoplasmic or mitochondrial translation. We therefore assessed protein aggregate formation in mitochondria after a mild heat shock in cells where mitochondrial or cytoplasmic protein synthesis was inhibited. To this end, we blocked mitochondrial translation with chloramphenicol (CAP) in cells grown on glucose, galactose, or glycerol (Figure 4A–D). However, under conditions that fully inhibited mitochondrial protein synthesis (Figure 4D), aggregate formation was not affected (Figure 4A–C). In contrast, inhibition of cytosolic translation using cycloheximide (CHX) strongly reduced aggregate formation under all three metabolic conditions (Figure 4E–H), despite that the heat shock should affect mature proteins in a similar way. Importantly, inhibition of cytosolic translation reduces levels of proteins that are being imported into mitochondria, indicating that particularly the newly imported proteins are prone to heat shock‐induced aggregation. To test whether indeed mature, steady‐state proteins would not aggregate under these conditions, we monitored the mitochondrial distribution of three different mScarlet‐fused proteins, namely Atp1 as a subunit of the ATP synthase, Cit1 as a soluble, monomeric TCA cycle enzyme, and Tim44, a peripheral membrane protein of the mitochondrial import machinery. As expected, these proteins did not aggregate upon a mild heat shock and remained evenly distributed throughout the mitochondrial network (Figure 4I).

**FIGURE 5:**
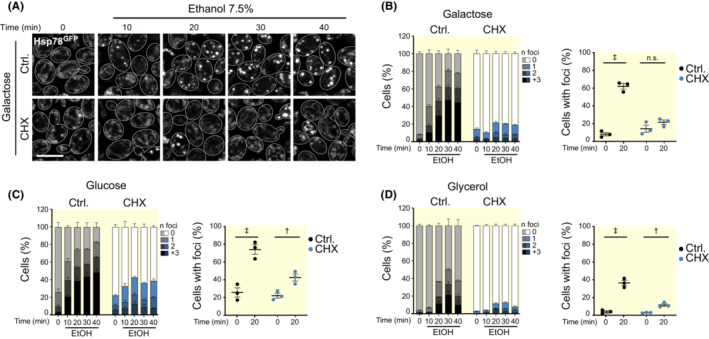
Effect of chemically induced denaturation on mitochondrial protein aggregation. (A) Confocal micrographs of Hsp78^GFP^ distribution in galactose‐grown cells upon addition of 7.5% ethanol (EtOH) in the presence of CHX (150 μg/mL) or solvent control analyzed every 10 min. Scale bar: 5 μm. (B) Quantification of the number of Hsp78^GFP^ aggregates per cell during the timeline shown in (A) and comparison of the number of cells with mitochondrial aggregates before and 20 min after treatment with EtOH in the presence of CHX or solvent control; *n* ≥ 121 cells. (C,D) Quantification as in (B) of cells grown in glucose (C) and glycerol (D); *n* ≥ 92 and *n* ≥ 125 cells, respectively (^n.s.^ *p* > 0.05; ^†^ *p* < 0.01; ^‡^ *p* < 0.001).

To substantiate the finding that particularly the newly imported proteins are prone to aggregation, we next chose to study chemically induced denaturation of proteins and how this was modulated by cytosolic protein synthesis. Previously, externally added ethanol at 7.5% was shown to provoke cytosolic aggregate formation in yeast that was sufficient to activate Hsf1 as a readout for protein denaturation.^33^ Thus, we exposed yeast cells to 7.5% ethanol and followed foci formation of Hsp78 over time. Robust formation of Hsp78 foci was observed after 10 min of ethanol exposure (Figure 5A–D). Similar to our findings with heat‐induced aggregation, respiring cells were more resistant to aggregate formation (Figure 5D). Importantly, inhibition of cytosolic translation via CHX prevented aggregation, and Hsp78 remained normally distributed (Figure 5B–D). These data confirm that newly synthesized and imported proteins are particularly vulnerable to stress‐induced aggregation. However, once assembled and properly folded, these proteins are stable and resistant to denaturation.

## 3 DISCUSSION

**FIGURE 6:**
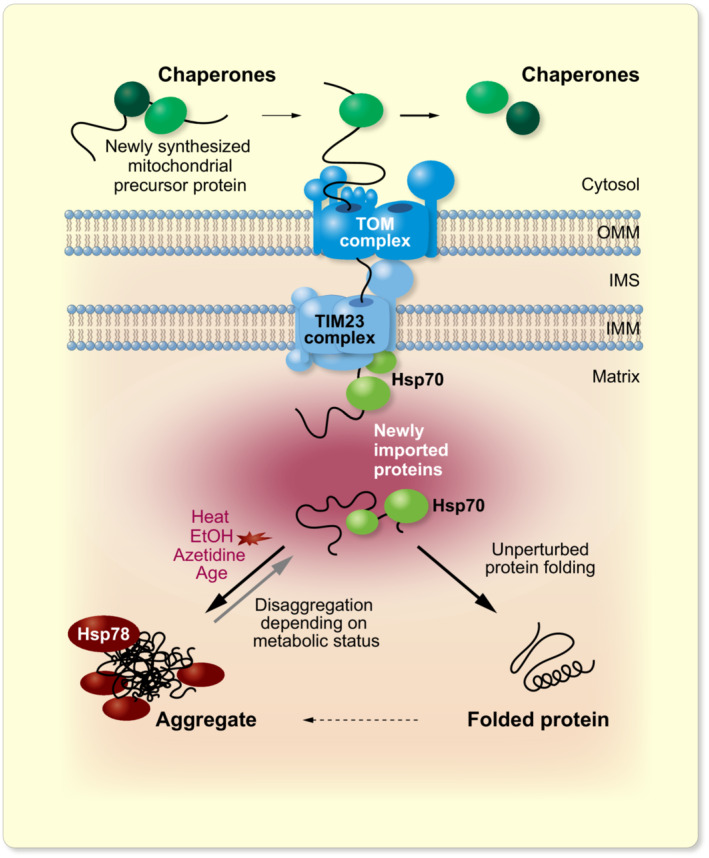
Newly imported mitochondrial proteins are particularly sensitive to aggregation. Schematic representation of the fate of newly synthesized mitochondrial proteins in cells exposed to stress. Under physiological conditions, imported proteins are able to fold into their native conformation, thus becoming stable and resistant to misfolding. In contrast, correct folding of newly imported proteins upon cellular stress is not as efficient and the not yet folded polypeptides are more prone to misfolding and aggregation. Handling and clearance of the mitochondrial aggregates strongly depends on the metabolic status of the cells by influencing both energy load as well as the expression levels of mitochondrial chaperones. Outer mitochondrial membrane (OMM), intermembrane space (IMS), and inner mitochondrial membrane (IMM).

Aggregation of proteins is a hallmark of proteostatic stress. Targeted protein sequestration in these aggregation sites is one strategy employed by cells to prevent misfolded proteins from damaging the proteome and occurs in many different organisms and under various conditions. In this work, we analyzed how protein aggregates in mitochondria are formed and dissolved upon stress, using heat‐induced or chemical denaturation as models. Our data point to a substantial modulation of aggregate handling depending on the metabolic state of the cell. While mitochondrial aggregates formed readily in fermenting cells, respiring cells or cells grown on galactose showed delayed aggregate formation and accelerated aggregate clearance (Figure 6).

These differences in aggregate clearance efficiency can likely be explained by two aspects, namely lower expression levels of mitochondrial PQC components during non‐stress conditions on fermentation and a higher energy status of the mitochondrial matrix during respiration. *HSP78* belongs to the class of genes regulated by Hsf1^12^ and, to a lesser extent, by Msn2/4, which are key transcription factors to mediate stress responses and the associated regulation of mitochondrial biogenesis. Thus, Hsp78 expression is comparably low during fermentation of glucose and can be substantially upregulated during heat shock and in the absence of glucose repression.^26^ This differential equipment of mitochondria with PQC components therefore is likely one key aspect that determines how efficiently unfolded proteins can be handled in mitochondria. Accordingly, lower levels of Hsp78 could explain why glucose‐grown cells show a rapid growth of aggregates and a delay in their clearance. Moreover, it is conceivable that the larger aggregates that form in these cells are more difficult to dissolve, as proteins sequestered inside these big aggregates might not be similarly accessible to Hsp78 as those found in smaller aggregates.

A second, likely at least as important aspect that determines aggregate handling is that the energy status of the mitochondrial matrix substantially differs between the different metabolic states. While mitochondrial ATP production is limited during glucose fermentation, fermentation of galactose or respiration leads to an activation of mitochondrial ATP synthesis via OXPHOS. This increased energy load should therefore facilitate the activity of ATP‐powered chaperones, including the chaperones HSP60, Ssc1 (the mitochondrial Hsp70‐homolog), and Hsp78. The well‐established interplay between Hsp78 and Ssc1 for refolding aggregated proteins^16^, ^34^, ^35^, ^36^ depends on a high‐energy state of mitochondria.^37^ In this cooperation, Ssc1 might weaken the interactions between aggregated proteins, allowing Hsp78 access to these proteins for subsequent extraction and unfolding, thereby permitting a new attempt for protein folding.^35^, ^37^

Aggregate handling in mitochondria occurs with similar mechanisms as in the cytosol. Here, thermal denaturation, chemical modifications of proteins, impairment of protein targeting to organelles, or deteriorated protein quality control leads to the formation of protein aggregates. Interestingly, aggregate formation in the cytosol also proceeds from the formation of small aggregates to their fusion into a large structure termed JUNQ or INQ, in case they cannot be resolved.^38^, ^39^, ^40^ In yeast, these aggregates are decorated by the Hsp78 homolog Hsp104 for extracting individual polypeptides to allow their refolding, and by small heat shock proteins that help to organize these aggregates. The activity of various chaperones then allows to extract polypeptides from aggregates to funnel them either into protein folding or degradation via the ubiquitin–proteasome system or autophagy. Aggregate formation in the cytosol or in the lumen of the ER elicits a specific transcriptional response, leading to the increased expression of compartment‐specific molecular chaperones.^41^, ^42^ Interestingly, both signaling pathways employ a mechanism, where availability of Hsp70 chaperones is used to activate transcription factors.^43^, ^44^ In the case of mitochondria, it is possible that non‐resolved aggregates sequester Hsp70, which in turn decreases its availability for de novo folding and protein import, which could decrease import efficiency and thus represent a signaling cue for a transcriptional response. However, whether and how a mitochondria‐specific unfolded protein response occurs in yeast is currently debated. Modulating mitochondrial translation efficiency,^45^ impairment of mitochondrial import,^46^, ^47^, ^48^ or impairment in processing of imported precursor proteins,^49^ all elicit a distinct transcriptional response that leads to the upregulation of mitochondrial biogenesis and a protective program to fortify the mitochondrial PQC. While this mitochondria‐to‐nucleus communication has been shown in animals to operate through a dedicated transcription factor^50^ or the integrated stress response,^51^ it is unclear how signals are conveyed in yeast. Given the power of yeast genetics, it should be feasible to construct strategies to unravel components of such a signaling pathway.

Previous studies used expression of aggregation prone proteins targeted to mitochondria to describe the large aggregate site by fluorescence microscopy that was termed ImiQ^15^ or DUMP,^14^ which persisted throughout the analyses. While these approaches allowed to shed light on the intracellular localization of these aggregates and their inheritance during cell division, it was not possible to follow dynamics of aggregate clearance. The propensity to successfully refold aggregated proteins could not only depend on the energy status of mitochondria and the biochemical equipment of mitochondria, but also on the chemical nature of the aggregated proteins. In cases, where unstable proteins are imported or where proteins are oxidatively damaged and hence unfold, it is perceivable that the proteins cannot be reactivated, and thus can be either stored in persisting, possibly inert, aggregates or terminally degraded by proteases. Indeed, mitochondria contain sets of proteases that are implicated in removing damaged proteins in the matrix including the LON protease homolog Pim1^52^, ^53^ or, in mammals, ClpP.^54^ Conversely, the absence of Pim1 leads to the formation of aggregates that were visualized as large, electron‐dense inclusions in the mitochondrial matrix.^55^ In this work, we chose to employ a mild heat shock as a model, because we expected that under these conditions cellular fitness and mitochondrial function could be maintained, which are prerequisites for efficient protein reactivation from aggregates. Indeed, we observed that aggregates could be dissolved in all metabolic states, but currently we cannot discriminate whether proteins in aggregates were reactivated or degraded. Establishing experimental approaches for analyzing these parameters will be an important aspect of future research.

Our data establish that newly imported proteins are particularly sensitive to aggregation (Figure 6). All matrix proteins carrying an import signal reach their destination through the import channel found in the TIM23 complex, where Tim44 mediates an interaction with Hsp70 chaperones.^56^ Once the new proteins are fully translocated, folding can be finalized either spontaneously or through the activities of HSP60 and Hsp70 chaperones.^57^ Importantly, in cases where proteolytic removal of the targeting signal is impaired, the precursor proteins fail to fold and form aggregates in the matrix.^49^ Moreover, proteins that are subunits of complexes need to assemble. A recent study estimated that almost all mitochondrial proteins participate in at least one protein complex.^58^ It is conceivable that in the states of their biogenesis, preceding the final fold and assembly, proteins are particularly sensitive to misfolding and aggregate formation. However, it was surprising to see that inhibition of cytosolic translation and therefore disruption in the supply of mitochondrial precursor proteins for import substantially suppressed the formation of mitochondrial aggregates. This suggests that aggregate formation depends on the bulk of newly synthesized proteins that are imported in the time course of the experiment. However, cytoplasmic aggregate formation^59^ and Hsf1 activation^33^ depend on ongoing protein synthesis, indicating that the vulnerability of not fully folded proteins for misfolding during their biogenesis could be a universal aspect of aggregate formation.

## 4 MATERIALS AND METHODS

### 4.1 Yeast strains and culturing conditions

All *Saccharomyces cerevisiae* strains used in this study are derived from the parental strain BY4742 (*MATα his3*Δ*1 leu2*Δ*0 lys2*Δ*0 ura3*Δ*0*), except for transmission electron microscopy, where the strain BY4741 (*MATa his3*Δ*1 leu2*Δ*0 met15*Δ*0 ura3*Δ*0*) was used. Both strains were obtained from Euroscarf. Deletion or genomic tagging of genes has been performed as described previously.^60^, ^61^ Yeast strains, primers used for the genomic modifications, and plasmids are listed in Supporting Information Tables S1–S3, respectively.

Yeast cells were grown in rich media YP (2% peptone, 1% yeast extract) supplemented with 2% glucose, 2% galactose, or 2% glycerol. For the heat shock experiments, cells were inoculated to 0.3 OD_600_ and incubated for 6 h at 28°C, 145 rpm in baffled flasks prior to heat stress. For in vivo radioactive labeling, cells were grown in synthetic minimum media (1.7 g/L yeast nitrogen base, 5 g/L (NH_4_)_2_SO_4_) supplemented with 2% galactose and essential amino acids (20 mg/L adenine, 20 mg/L uracil, 30 mg/L leucine, 30 mg/L lysine, 15 mg/L tryptophan, 15 mg/L histidine, 20 mg/L arginine).

### 4.2 Drop dilution assay

In a 96‐well plate, 1 × 10^7^ cells were harvested and diluted in 5 serial dilutions of 1/5. Of each dilution, 3 μL were spotted onto glucose‐ and glycerol‐containing plates and incubated for up to 4 days at 30 and 37°C.

### 4.3 Mitochondria isolation and native‐PAGE

Mitochondria (100 μg), isolated as previously described,^45^ were lysed (50 mM Bis‐Tris, pH 7.2, 50 mM KCl, 2 mM amino hexanoic acid, 1 mM EDTA, 1× complete, 1 mM PMSF, 2% digitonin, 12% glycerol) for 10 min on ice. After a clarification spin (16 000 *g* for 10 min) sample additive (5% G‐250) was added and the lysates were separated on a native 3%–12% Bis‐Tris gel (NativePAGE, Thermo Fisher Scientific). Gels were stained with Coomassie brilliant blue and band intensities were quantified using Image Lab software (Bio‐Rad).

### 4.4 In vivo labeling of newly synthesized mitochondrial proteins

Radiolabeling of mitochondrial translation products was done as described previously.^62^ In brief, 2 OD_600_ of cells were harvested after 6 h of growth on minimum media and incubated for 10 min at 30°C, shaking at 750 rpm. Chloramphenicol (CAP; 1 mg/mL) or the corresponding ethanol control was added to the cells and incubated for 15 min, prior to the start of the labeling. Next, cycloheximide was added to a final concentration of 150 μg/mL to inhibit cytosolic protein synthesis and incubated for 2 min, after which [^35^S]‐methionine was added to the cells for 10 min. The labeling was stopped by the addition of the Stop‐mix (1.85 M NaOH, 1 M β‐mercaptoethanol, 20 mM PMSF) and 13 mM methionine. Samples were incubated on ice for 10 min, followed by protein extraction and SDS‐PAGE as described next. Radiolabeled proteins were detected in a Fujifilm FLA‐9000 scanner (Fujifilm, Japan).

### 4.5 Immunoblotting

Proteins were extracted by incubation with 14% TCA overnight at −20°C followed by two high‐speed centrifugations (20 000 *g*, 30 min) and washed with ice‐cold acetone. Protein pellets were resuspended in sample buffer (100 mM Tris, pH 6.8, 4% SDS, 20% glycerol, 0.2% bromophenol blue, 100 mM DTT) and heated at 95°C for 3 min.

Samples were separated on 16% acrylamide/0.2% *bis*‐acrylamide gels using Tris‐glycine running buffer (25 mM Tris, 192 mM glycine, 0.1% SDS) and transferred on to 0.2 μm nitrocellulose membranes. Membranes were briefly stained with Ponceau S solution (0.2% Ponceau S, 5% acetic acid) and then blocked in 5% milk/TBS (0.5 M Tris–HCl, 1.5 M NaCl, pH 7.4) for 1 h. Primary antibodies against GFP (1:2500, Roche, 1181446001) and Tom22 (1:1000, gift from N. Vögtle lab) and the secondary antibodies anti‐mouse (Sigma‐Aldrich A9044‐2ML) and anti‐rabbit (Sigma‐Aldrich A0545‐1ML) were used. All signals were detected with ChemiDoc XRS+ imaging System (Bio‐Rad) and quantified with Image Lab software (Bio‐Rad). Ponceau S signal was used as a loading control.

### 4.6 Data mining and visualization

Deposited data from Jaworek et al.^27^ were reanalyzed. To that end, the abundance of proteins (present in at least two of three biological replicates) identified in mitochondrial aggregates during heat stress (42°C) were compared with control conditions (25°C). Aggregating proteins from the mitochondrial matrix with a fold change of 2 or higher (visualized as node color) are depicted. These hits were compared to mitochondrial matrix proteins interacting with Hsp78 during heat stress (42°C). Here, the abundance of proteins co‐purified with Hsp78‐HIS were compared to untagged control samples. Mitochondrial matrix proteins present in at least 2 out of 3 biological replicates with a fold change of 1.5 or higher were used for visualization in CytoScape (v 3.9.1).

### 4.7 Confocal microscopy and quantification

Cells were harvested at the indicated time points and fixed with 4% paraformaldehyde (PFA) in phosphate‐buffered saline (PBS), pH 7 prior to image acquisition. For fixation, cell pellets were resuspended in 4% PFA and incubated at room temperature for 30 min followed by two washes with PBS. Cell pellets were finally resuspended and stored in PBS at 4°C until imaging.

For image acquisition, cells were immobilized on agar slides (3% agarose in PBS) and images were acquired on a LSM700 confocal microscope (Zeiss), equipped with a Plan/Apochromat 63×/1.40 oil DIC M27 objective. Excitation/emission wavelengths were 488 nm/300–550 nm for GFP and 555 nm/566–800 nm for mScarlet. *Z* stacks were acquired with slice thickness of 0.34 μm.

Image post‐processing and quantification was carried out with the open‐source software Fiji.^63^ Representative micrographs are maximum intensity *Z* projections (ZPMI) treated with a Gaussian blur (*σ* = 1) to reduce noise. Brightness and contrast were adjusted accordingly to ensure the visualization of the mitochondrial network. Cell outlines were drawn using the brightfield image as reference. For the quantification of the number of aggregates per cell, cells were segmented from Brightfield images using YeastMate^64^ and aggregates were segmented from the Hsp78^GFP^ ZPMI micrographs based on signal intensity. Thresholds for aggregate segmentation were manually set taking the heat shock time points as reference or the 20 min ethanol treatment (for Figure 5). Quantification of the number of aggregates per cell was done using the Speckle inspector tool from the Biovoxxel Toolbox (https://doi.org/10.5281/zenodo.5986130), for which cells were selected as primary objects and aggregates, filtered by size (5‐infinite px) and circularity (0.7‐1.0), were selected as secondary objects.

### 4.8 Transmission electron microscopy

For transmission electron microscopy, cells were collected during exponential growth (8 h after inoculation), during stationary phase (48 h after inoculation), upon treatment with azetidine (1 mg/mL for 90 min in exponential phase), as well as upon heat stress (45 min at 38°C or 30 min at 42°C during exponential phase), and samples were prepared as described previously.^65^, ^66^, ^67^ In brief, yeast cells were cryoimmobilized using a Wohlwend Compact 03 high‐pressure freezer (M. Wohlwend GmbH). The subsequent freeze substitution was performed in a solution of 2% uranyl acetate dissolved in 10% methanol and 90% acetone at −90°C for 1 h in a Leica EM AFS2 (Leica Microsystems). Samples were then heated at 2.9°C per hour to −50°C. Meanwhile, two washes in acetone were performed. Samples were infiltrated with increasing amounts of Lowicryl HM20 resin (Polysciences, 15924‐1) in acetone (1:4, 2:3, 1:1, 4:1, and 100% 3×) at −50°C. Each infiltration step lasted 2 h. Polymerization was induced with UV light for 72 h at −50°C followed by 24 h slowly warming up to room temperature. Samples were kept apart during this process using the Leica reagent bath with a flow‐through ring. Thin sections (70 nm) were cut using a diamond knife (45°, Diatome) on a Reichert‐Jung Ultracut E Ultramicrotome (C. Reichert). Uranyl acetate (2%) and Reynold's lead citrate^68^ were used for on‐section contrast staining. Images were acquired on a 120 kV Tecnai T12 electron microscope equipped with a Ceta CMOS 16M camera (FEI Co.).

### 4.9 Statistical analysis

Data are presented as line graphs, column graphs, or dot plots with error bars representing the standard error of the mean (SEM). Statistical analysis of the data was carried out using graphpad prism (v8.0). For the line graph in Figure 2C, data are presented as means with SEM and the areas under the curve were compared with a one‐way ANOVA with Tukey's post hoc test. A two‐way ANOVA with Sidak's post hoc test was used to compare the data on the dot plots presented in Figures 4C,G and 5B–D and in the column graph presented in Figure 2E,F.

## CONFLICT OF INTEREST STATEMENT

The authors declare no conflict of interest.

## Supporting information


Supplementary Tables S1‐S3.
