# Supplementary material for: Newly imported proteins in mitochondria are particularly sensitive to aggregation
Source: Acta Physiol (Oxf). 2023 Jun 1;238(3):e13985. doi: 10.1111/apha.13985 (PMC10909475; doi:10.1111/apha.13985)
Supplement: Supplementary file 1 — Supplementary Tables S1‐S3. [file APHA-238-e13985-s001.pdf]

Supplementary Table S1. Yeast strains used in this study.

**Strain**	Genotype	Source
BY4741	MATa his3Δ1 leu2Δ0 met15Δ0 ura3Δ0	Euroscarf
BY4742	MATα, his3Δ1, leu2 0, lys2Δ0, ura3Δ0	Euroscarf
BY4742 Hsp78^{yeGFP}	BY4742 *HSP78*-yeGFP-hphNT1	This study
BY4742 Hsp78^{yeGFP} Cit1^{mScarlet}	BY4742 *HSP78*-yeGFP-hphNT1, *CIT1*-mScarlet-kanMX	This study
BY4742 Hsp78^{yeGFP} Atp1^{mScarlet}	BY4742 *HSP78*-yeGFP-hphNT1, *ATP1*-mScarlet-kanMX	This study
BY4742 Hsp78^{yeGFP} Tim44^{mScarlet}	BY4742 *HSP78*-yeGFP-hphNT1, *TIM44*-mScarlet-kanMX	This study
BY4742 Hsp78yeGFP Δ*pdr5*	BY4742 *HSP78*-yeGFP-hphNT1, *pdr5*Δ::natNT2	This study
BY4742 Δ*hsp78*	BY4742 *hsp78*Δ::kanMX	This study
BY4742 Δ*fmc1*	BY4742 *fmc1*Δ::HIS3	This study
BY4742 Δ*hsp78* Δ*fmc1*	BY4742 *hsp78*Δ::kanMX, *fmc1*Δ::HIS3	This study

Supplementary Table S2. Oligonucleotides used for gene deletion and genomic tagging.

**Modification**	**Oligonucleotides**	**PCR template**
C-terminal tagging of HSP78	5'- GCTACCAAATCATGAAGAAGGCGAAGTTGTTGAAGAGGAAG CTGAAAAGCGTACGCTGCAGGTCGAC -3'<br>5'- ATGTATTTTCTTGGGAATTTATTATTCATAAACCGCTTGTGCAG TTAATCGATGAATTCGAGCTCG -3'	pYM25-yeGFP-hphNT1<br>(Janke et al., 2004)
Control PCR *HSP78* tagging	5'- ACGCTTAAAACAGAACTACGAG -3'<br>5'- CGAGCTCGAATTCATCGAT -3'	
Deletion of *HSP78*	5'- CTATTCCAGGAACCCTGAAACAAGCGAGTGAAAATCTTTCAAG GTTAAATCAGCTGAAGCTTCGTACGC -3'<br>5'- ATGTATTTTCTTGGGAATTTATTATTCATAAACCGCTTGTGCAG GCATAGGCCACTAGTGGATCTG -3'	pUG6- kanMX (Gueldener et al. 2002)
Control PCR *HSP78* deletion	5'- CCTGGAGGAAATAGTAGGGA- 3'<br>5'- GCGTACGAAGCTTCAGCTG -3'	
C-terminal tagging of *CIT1*	5'- CTCCACCGAAAAATACAAGGAGTTGGTAAAGAAAATCGAAAGT AAGAACCGTACGCTGCAGGTCGAC -3'<br>5'- CATAGGGGACTCAAAGCGTAAAAATCATGAAGTCATAGCCATT TCCTTATGATCGATGAATTCGAGCTCG- 3'	pSB90-mScarlet-kanMX<br>(This study)
Control PCR *CIT1* tagging	5'- CATTGATATCATTTGCCAAG -3'<br>5'- GTCGACCTGCAGCGTACG -3'	
C-terminal tagging of *ATP1*	5'- CATTTCTTTTTGAGACGTACCTTATATTCATTTTTATTTTTTTAG TTCACATTAATCGATGAATTCGAGCTCG -3'<br>5'- GTTGGCATCTCTAAAGAGTGCTACTGAATCATTTGTTGCCACT TTTCGTACGCTGCAGGTCGAC -3'	pSB90-mScarlet-kanMX<br>(This study)
Control PCR *ATP1* tagging	5'- CCTGCACCTATAATGGCCAC -3'<br>5'- CGAGCTCGAATTCATCGAT -3'	

C-terminal tagging of *TIM44*	5'- GAATAGGAAGGAAAAGGAAAAGAAAACAAAAGAGTACATCGA AACCAATCAATCGATGAATTCGAGCTCG -3'<br>5'- GGGTGGAAGATCTTGGAGTTTGTGCGCGGGGGTTCTAGACAA TTCACCCGTACGCTGCAGGTCGAC -3'	pSB90-mScarlet-kanMX<br>(This study)
Control PCR *TIM44* tagging	5'- CATGACTCTACGGAGAGAATTG -3'<br>5'- CGAGCTCGAATTCATCGAT -3'	
Deletion of *FMC1*	5'- GATAACAAAAAGCTGAAAAGAGAGAGAAAGAGAGAGTGCCAG GGAAATGCGTACGCTGCAGGTCGAC -3'<br>5'- CATCAAATATATATATATACTTATTTACAAAACTCTGATATTATA AATTGATTAGATACTAATCGATGAATTCGAGCTCG -3'	pFA6a-HIS3MX (Janke et al., 2004)
Control PCR *FMC1* deletion	5'- GTGCGATGTTCCTGAATTTTG -3'<br>5'- GTCGACCTGCAGCGTACG -3'	
Deletion of *PDR5*	5'- CCCTTTTAAGTTTTCGTATCCGCTCGTTCGAAAGACTTTAGAC AAAAATGCGTACGCTGCAGGTCGAC -3'<br>5'- GTCCATCTTGGTAAGTTTCTTTTCTTAACCAAATTCAAAATTCT ATTAATCGATGAATTCGAGCTCG -3'	pFA6a-natNT2 (Janke et al., 2004)
Control PCR *PDR5* deletion	5'- CGAACGTTCGCATTCTGCGC -3'<br>5'- GTCGACCTGCAGCGTACG -3'	

Supplementary Table S3. Plasmid generated in this study.

Plasmid	Cloning strategy	Template
pSB90	42 bp linker + mScarlet introduced with BsWI and BamHI into pYM27 (Janke et al., 2004)<br>Primers:<br>5’-TAAGCACGTACGCTGCAGGTCGACGGATCAGGAGCAGGTGCTGGTGCTGG TGCTGGAGCAATTCTGATGGTGAGCAAGGGCGAGG -3’<br>5’- ATCTGGATCCTTATTTATATAATTCATCCATACCACCAG -3’	pDRF1-GW mScarlet-T2A-mTurquoise2<br>(Addgene)
